# Caffeine supplementation is ergogenic in soccer players independent of cardiorespiratory or neuromuscular fitness levels

**DOI:** 10.1186/s12970-020-00360-x

**Published:** 2020-06-08

**Authors:** Andreas Apostolidis, Vassilis Mougios, Ilias Smilios, Maria Frangous, Marios Hadjicharalambous

**Affiliations:** 1 Human Performance Laboratory, Department of Life & Health Sciences, University of Nicosia, 46 Makedonitissas Ave., P.O. Box 24005, 1700 Nicosia, Cyprus; 2 Laboratory of Evaluation of Human Biological Performance, School of Physical Education & Sport Science, Aristotle University of Thessaloniki, Thessaloniki, Greece; 3 School of Physical Education & Sports Science, Democritus University of Thrace, Komotini, Greece; 4 Nicosia General Hospital, Nicosia, Cyprus

**Keywords:** Ergogenic aid, Endurance performance, Explosiveness, Biochemical responses

## Abstract

**Background:**

Equivocal findings examining the influence of caffeine on performance and biological responses to exercise may be due to inter-individual variability in cardiorespiratory or neuromuscular fitness. This study examined whether the effects of caffeine ingestion on exercise performance and biological responses to prolonged intermittent exercise to exhaustion depend on cardiorespiratory or neuromuscular fitness.

**Methods:**

Twenty male soccer players, separated according to either cardiorespiratory fitness (high vs medium) or neuromuscular fitness (high vs medium) underwent two trials simulating the cardiovascular demands of a soccer game to exhaustion on treadmill after ingesting either caffeine (6 mg∙kg^− 1^) or placebo. Physical performance, cardiorespiratory and metabolic parameters and blood metabolites were evaluated.

**Results:**

Time to exhaustion (719 ± 288 vs 469 ± 228 s), jump height (42.7 ± 4.2 vs 38.6 ± 4.4 cm), heart rate (163 ± 12 vs 157 ± 13 b**∙**min^− 1^), mean arterial blood pressure (98 ± 8 vs 92 ± 10 mmHg), plasma glucose (5.6 ± 0.7 vs 5.3 ± 0.6 mmol∙l^− 1^) and lactate (3.3 ± 1.2 vs 2.9 ± 1.2 mmol∙l^− 1^) were higher, while rating of perceived exertion (12.6 ± 1.7 vs 13.3 ± 1.6) was lower with caffeine vs placebo (*p* < 0.01), independent of cardiorespiratory or neuromuscular fitness level. Reaction time; plasma glycerol, non-esterified fatty acids and epinephrine; carbohydrate and fat oxidation rates; and energy expenditure were not affected by caffeine (*p* > 0.05).

**Conclusions:**

Caffeine was effective in improving endurance and neuromuscular performance in athletes with either high or medium cardiorespiratory and neuromuscular fitness. Cardiorespiratory and neuromuscular fitness do not appear to modulate the ergogenic effects of caffeine supplementation in well-trained athletes.

## Introduction

Over the past three decades, caffeine has been one of the most popular ergogenic aids, used by athletes performing a variety of endurance and speed/power sports [1]. Nevertheless, the precise metabolic and/or neural mechanisms of caffeine’s ergogenicity remain unknown [2, 3]. Earlier studies suggested that caffeine may enhance fat oxidation and improve endurance performance by promoting intramuscular carbohydrate sparing [4], findings that are supported by more recent reports [5, 6]. Other studies, however, have failed to support a carbohydrate sparing effect of caffeine [7] or a positive effect on endurance performance [8, 9]. In well-controlled studies in which endurance performance improved following caffeine ingestion, fuel oxidation [10] and physiological responses to exercise were not significantly altered by caffeine [11], which questions even further the existence of a metabolic basis for the ergogenic effect of caffeine despite the finding that this effect increases with event duration [12]. Although an expectancy effect on physical performance cannot be excluded, it might be that caffeine’s ergogenicity can be best explained by a reduction in rating of perceived exertion (RPE), probably due to adenosine antagonism in the central nervous system [12].

Caffeine has been found to increase anaerobic performance [13] and Wingate peak and mean power [14, 15], as well as indices of neuromuscular performance such as maximal muscle strength and endurance of the lower body, and vertical jump performance [3, 15]. These effects occurred in settings where neither glycogen metabolism is the primary determinant of muscular performance, nor is glycogen depletion a cause of fatigue. Improved neuromuscular performance could be attributed to improved strength and motor-unit recruitment rate [16] and/or to increased voluntary activation [2]. Others, however, have not observed any ergogenic effect of caffeine on lower body maximal strength or endurance [14, 17, 18] or on upper-body maximal muscle strength, power or endurance [3].

Considerable biological and methodological variability between studies, including exercise type, participants’ characteristics (e.g., age, sex, training experience, training status), caffeine dosage [2, 15, 18], and genetic predisposition [19], might be responsible for the discrepancies regarding caffeine’s action. An aspect, however, that has not been adequately examined and may influence the effects of caffeine on exercise performance and metabolism is inter-individual variability in training status or fitness level. Collomp et al. [20] found that anaerobic capacity (swimming speed during two 100 m trials) improved with caffeine in highly trained swimmers but not in untrained, occasional swimmers. Brooks et al. [21], however, did not observe any effect of caffeine on another physical performance parameter, one-repetition maximum squat performance, in either resistance trained or untrained individuals. Astorino et al. [5] evaluated the ergogenicity of caffeine during an endurance task (10 km cycling time trial lasting about 18 min) and found an improvement in endurance-trained (mean VO_2_max of 57.5 ml∙kg^− 1^∙min^− 1^) but not in recreationally active participants (mean VO_2_max of 46.5 ml∙kg^− 1^∙min^− 1^). On the other hand, Shen et al. [12], in a recent meta-analysis, found no significant association of VO_2_max with the magnitude of caffeine’s ergogenicity.

Consequently, further studies are merited to elucidate the psychophysiological and performance effects of caffeine during exercise in persons of different endurance or neuromuscular fitness levels. The use of homogeneous groups of athletes in terms of training status may assist to better examine this question. The purpose, therefore, of the present study was to examine whether the effects of caffeine supplementation on biological responses and exercise performance depend on levels of cardiorespiratory or neuromuscular fitness by using the same protocol and study participants as in our previous study [10]. We hypothesized that caffeine ingestion would provoke dissimilar biological responses to exercise and have dissimilar performance effects between athletes with different cardiorespiratory and/or neuromuscular fitness levels.

## Methods

### Participants

Twenty healthy male soccer players took part in the study voluntarily. The study was approved by the Cyprus National Bioethics Committee (ΕΕΒΚ/ΕP/2015/20) and conformed to the Code of Ethics of the World Medical Association (Declaration of Helsinki). The participants had previous professional or semi-professional soccer experience of at least 5 years with regular training and participation in official national league soccer games. Following explanation of tests and procedures, as well as the nature, benefits and risks of the study during a preliminary session, the participants gave their written consent, after which medical history, lifestyle, and caffeine consumption questionnaires were completed. Anthropometric characteristics and VO_2_max were measured as described [10].

### Experimental design

Details of the test procedure have been published [10]. Briefly, the participants underwent two identical exercise trials, separated by at least 4 days, after consuming either 6 mg∙kg^− 1^ of caffeine or placebo in a crossover, double-blind, and counterbalanced manner. Each trial included three 22.5 min periods (each equal to one quarter of a 90 min soccer game) of running at variable speeds on treadmill, simulating the cardiovascular demands of a soccer game on the basis of Drust et al. [22]. These were then followed by a period of running to exhaustion at a constant speed corresponding to 75% of each participant’s VO_2_max. The three intervals between the four periods lasted, in sequence, 5 min, 15 min (corresponding to the interval between halves in a soccer game) and 5 min; these intervals served for resting and measurements.

Mean arterial blood pressure (MAP) measurement and blood sampling were performed before each trial, during each interval, and at exhaustion. Countermovement jump (CMJ) height and reaction time (RT) were measured before each trial and during each interval (not at exhaustion). Heart rate (HR) was continuously recorded during the trials. RPE was determined at the beginning, middle and end of each of the first three periods, as well as at the beginning and third minute of the fourth period. Expiratory gases were measured during the first 5 min of the fourth period. O_2_ uptake and CO_2_ production data were used to measure respiratory exchange ratio (RER), energy expenditure and substrate oxidation rates. Blood was used to prepare EDTA-plasma, in which glucose, lactate, glycerol, non-esterified fatty acids (NEFA), and epinephrine were measured.

### Classification according to cardiorespiratory or neuromuscular fitness

The study sample was dichotomized in two ways: on the basis of VO_2_max and on the basis of the mean CMJ height before the two trials. The 10 participants with the highest VO_2_max values formed the high cardiorespiratory fitness (HCF) group, while the 10 participants with the lowest VO_2_max values formed the medium cardiorespiratory fitness (MCF) group. Likewise, the 10 participants with the highest CMJ values formed the high neuromuscular fitness (HNF) group, while the 10 participants with the lowest CMJ values formed the medium neuromuscular fitness (MNF) group.

### Statistical analysis

Data are expressed as the mean and SD or as median and range, depending on whether the distribution did not or did differ significantly from normal, respectively, according to the Shapiro-Wilk test. Characteristics of the groups were compared by Student’s t test or Mann-Whitney *U* test, as appropriate. Data on time to exhaustion, RER, energy expenditure, and fuel oxidation were analyzed by two-way ANOVA (group x treatment), while data on CMJ, RT, MAP, HR, RPE, glucose, lactate, glycerol, NEFA, and epinephrine were analyzed by three-way ANOVA (group x time x treatment) with repeated measures on time and treatment and Sidak correction on post-hoc comparisons. Mauchly’s test of sphericity was performed for all test variables, and Greenhouse-Geisser correction for within-subject effects was employed in cases where the assumption of sphericity was violated. Effect sizes (ES) were estimated by calculating partial eta squared and were classified as small (0.01 to 0.058), medium (0.059 to 0.137) or large (0.138 or higher), according to Cohen [23]. To control for body mass and body fat, data were also analyzed by ANCOVA. For all statistical analyses a value of *p* ≤ 0.05 was considered significant. Analyses were performed in SPSS, version 22.

## Results

### Group characteristics

The anthropometric and physiological characteristics of the participants, divided according to cardiorespiratory or neuromuscular fitness, are presented in Table 1. Groups differed significantly in the selection parameter (VO_2_max or CMJ) by design. Additionally, the high-fitness groups had significantly lower percentage body fat that the corresponding medium-fitness groups, and the HNF group had significantly higher VO_2_max than the MNF group. Groups did not differ significantly in habitual daily caffeine consumption, which was moderate (with an overall median of 0.9 mg∙kg^− 1^) and considerably lower than the experimental dose of 6 mg∙kg^− 1^.

**Table 1:** Characteristics of participants with high (HCF) or medium cardiorespiratory fitness (MCF); and with high (HNF) or medium neuromuscular fitness (MNF)

Group	Age (years)	Body height (m)	Body mass (kg)	Body fat (%)	VO_2_max (ml∙kg^− 1^∙min^− 1^)	CMJ (cm)	Daily caffeine consumption (mg)	Daily caffeine consumption (mg∙kg^− 1^)
HCF (*n* = 10)	21 ± 4	1.78 ± 0.05	71.93 ± 6.30	9.61 ± 2.58*	64.35 ± 2.37*	40.23 ± 1.98	65 (17–373)	0.9 (0.2–4.6)
MCF (*n* = 10)	22 ± 4	1.78 ± 0.07	76.39 ± 8.68	13.31 ± 3.73	57.16 ± 2.16	39.39 ± 6.63	120 (0–351)	1.6 (0–3.9)
HNF (*n* = 10)	21 ± 3	1.77 ± 0.05	71.95 ± 6.45	9.76 ± 2.65*	61.72 ± 4.69*	43.30 ± 3.28*	66 (15–375)	0.9 (0.2–4.6)
MNF (*n* = 10)	22 ± 4	1.80 ± 0.06	76.37 ± 8.58	13.16 ± 3.84	59.79 ± 3.87	36.32 ± 3.22	104 (0–351)	1.5 (0–3.9)

Data are presented as mean ± SD or median (range)

*Significant difference from the corresponding medium-fitness group (*p* < 0.05)

### Time to exhaustion

**Fig. 1:**
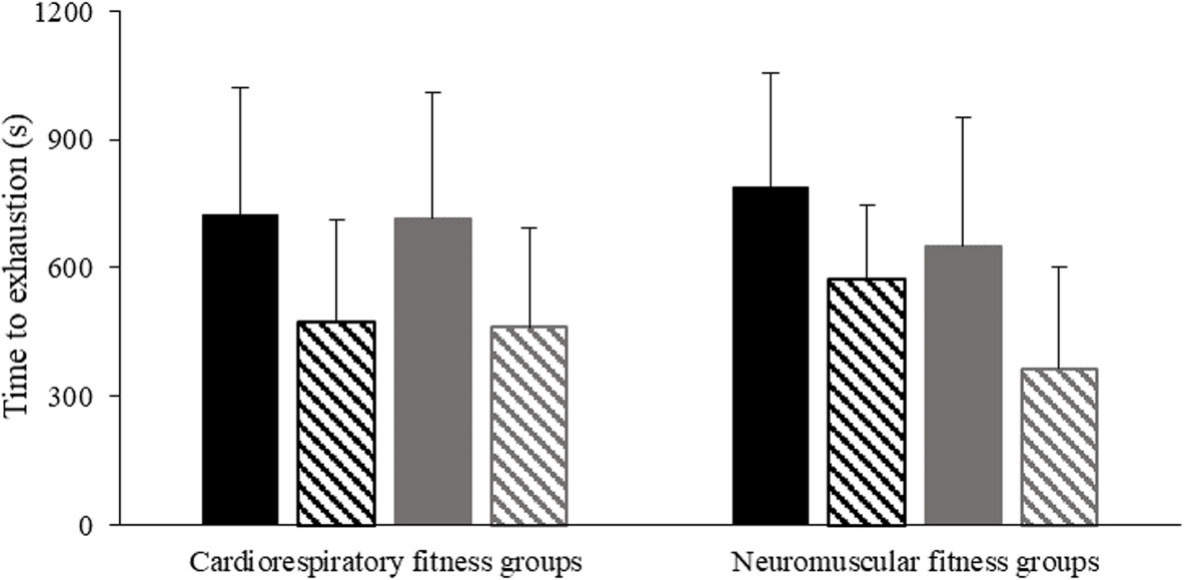
Means and SD of time to exhaustion with caffeine (solid bars) and placebo (hatched bars). Participants were divided according to cardiorespiratory (left) or neuromuscular fitness (right), with high groups shown in black and medium groups shown in grey. There was a significant treatment effect (*p* < 0.001)

There was a significant main effect of treatment (caffeine vs placebo) on time to exhaustion (Fig. 1) regardless of whether participants were classified based on cardiorespiratory fitness [F(1, 18) = 29.15, *p* < 0.001, ES = 0.561] or on neuromuscular fitness [F(1, 18) = 23.59, *p* < 0.001, ES = 0.567]. Time to exhaustion was longer with caffeine compared with placebo (719 ± 288 vs 469 ± 228 s). However, time to exhaustion was not different between groups, and there was no treatment-by-group interaction (*p* > 0.05).

Three of the participants who performed the caffeine trial after the placebo trial noticed their longer times to exhaustion and guessed they had taken caffeine. None of the other participants made any presumption related to what they were taking.

### Jump performance and reaction time

**Fig. 2:**
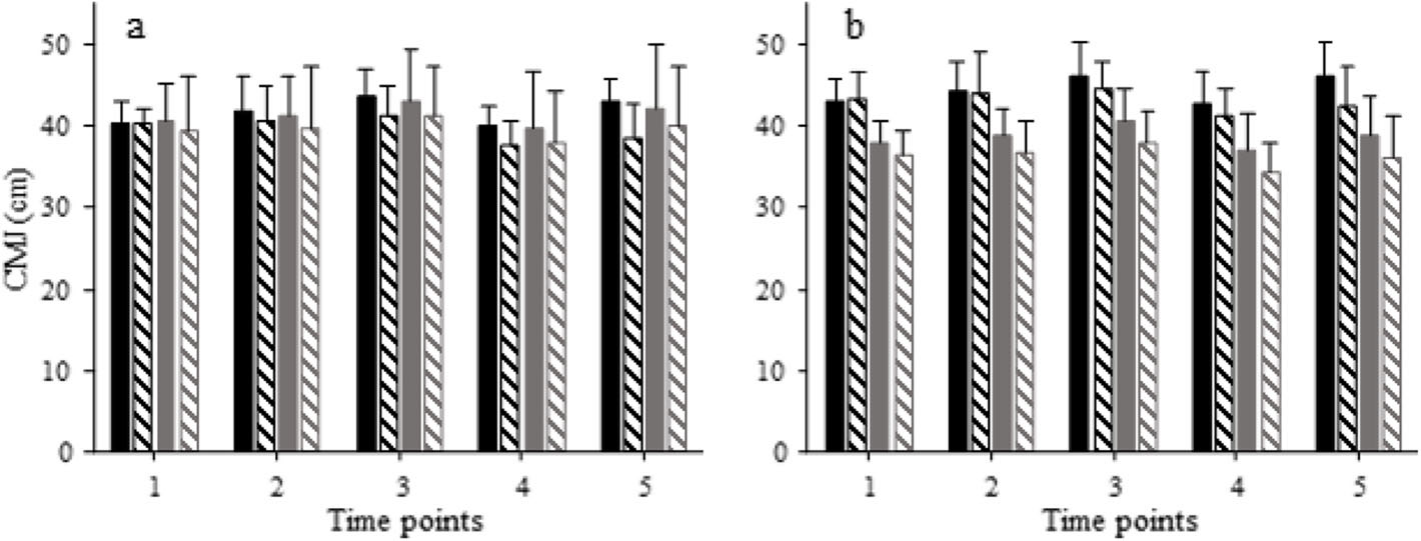
Means and SD of countermovement jump height with caffeine (solid bars) and placebo (hatched bars). Participants were divided according to cardiorespiratory (**a**) or neuromuscular fitness (**b**), with high groups shown in black and medium groups shown in grey. One to 5 correspond to time points as follows: 1, before the start of the trial; 2, between the 1st and 2nd periods; 3, immediately after the end of the 2nd period; 4, immediately before the start of the 3rd period; 5: between the 3rd and 4th periods. There were significant treatment and time effects in both a and b, as well as a significant group effect (by design) in b (*p* < 0.05)

There were significant treatment [F(1, 18) = 22.84, *p* < 0.001, ES = 0.559] and time [F(4, 72) = 16.13, *p* < 0.001, ES = 0.473] main effects on CMJ (Fig. 2a), as well as a time main effect on RT [F(4, 72) = 3.15, *p* = 0.02, ES = 0.149, data not shown], while there was no significant group effect or interaction (*p* > 0.05) for the cardiorespiratory fitness classification. CMJ was higher with caffeine compared to placebo (42.7 ± 4.2 vs 38.6 ± 4.4 cm overall).

For the neuromuscular fitness classification, (Fig. 2b) CMJ exhibited significant main effects of group [F(1, 18) = 16.91, *p* < 0.001, ES = 0.484, as expected, since groups were defined on the basis of CM], treatment [F(1, 18) = 25.38, *p* < 0.001, ES = 0.585], and time [F(4, 72) = 16.17, *p* < 0.001, ES = 0.473], while there was no interaction (*p* > 0.05). There was no significant main effect or interaction in RT (*p* > 0.05, data not shown).

### Cardiovascular responses

When the data were analyzed based on cardiorespiratory fitness level, there was a treatment [F(1, 18) = 12.85, *p* = 0.002, ES = 0.417] and a time [F(2, 41) = 17.68, *p* < 0.001, ES = 0.495] main effect on MAP (Table 2). MAP was higher with caffeine compared to placebo (98 ± 8 vs 92 ± 10 mmHg overall). Similarly, when the data were analyzed based on for neuromuscular fitness level (Table 2), there was a treatment [F(1, 18) = 12.81, *p* = 0.002, ES = 0.416] and a time [F(2, 44) = 18.51, *p* < 0.001, ES = 0.507] main effect on MAP. There was no significant group effect or interaction for either classification (*p* > 0.05).

**Table 2:** Mean arterial pressure for the cardiorespiratory and neuromuscular fitness classifications (mm Hg, mean ± SD)

Classification	Treatment	Group	Time points						
			1^a^	2	3	4	5	6	7
Cardiorespiratory fitness*^†^	Caffeine	HCF	86.22 ± 9.17	93.67 ± 7.29	108.70 ± 11.04	101.17 ± 3.37	90.53 ± 4.30	104.07 ± 8.43	102.63 ± 8.98
		MCF	92.52 ± 7.30	95.00 ± 7.41	102.00 ± 8.96	98.00 ± 5.58	93.83 ± 6.46	100.00 ± 8.93	105.00 ± 9.66
	Placebo	HCF	85.53 ± 11.61	85.40 ± 5.90	98.07 ± 6.71	89.30 ± 10.79	74.70 ± 27.21	94.23 ± 10.21	98.90 ± 11.06
		MCF	91.28 ± 5.60	91.02 ± 4.29	101.57 ± 4.47	97.40 ± 5.71	82.00 ± 29.04	99.07 ± 6.30	100.37 ± 4.95
Neuromuscular fitness*^†^	Caffeine	HNF	90.57 ± 9.31	93.27 ± 7.74	104.40 ± 11.05	99.23 ± 5.56	91.27 ± 6.12	101.93 ± 8.96	101.13 ± 8.56
		MNF	88.17 ± 8.34	95.40 ± 6.84	106.30 ± 10.15	99.93 ± 4.11	93.10 ± 5.20	102.13 ± 8.93	106.50 ± 9.36
	Placebo	HNF	87.73 ± 12.20	87.18 ± 5.81	98.80 ± 5.74	90.20 ± 11.60	67.40 ± 36.21	93.07 ± 9.42	96.73 ± 10.15
		MNF	89.08 ± 5.89	89.23 ± 5.89	100.83 ± 6.04	96.50 ± 5.37	89.30 ± 5.95	100.23 ± 6.30	102.53 ± 5.12

^a^1 to 7 correspond to time points as follows: 1, 60 min before the start of the trial; 2, immediately before the start of the trial; 3, between the 1st and 2nd periods; 4, immediately after the end of the 2nd period; 5, immediately before the start of the 3rd period; 6: between the 3rd and 4th periods; 7: exhaustion

* Significant main effect of treatment (*p* < 0.01)

^†^ Significant main effect of time (*p* < 0.001)

Average HR during each of the four periods of the treadmill protocol (Table 3) showed significant main effects of treatment [F(1, 16) = 17.77, *p* < 0.001, ES = 0.526] and time [F(2, 32) = 12.57, *p* < 0.001 ES = 0.440], as well as an interaction between treatment and time [F(3, 48) = 3.06, *p* = 0.04, ES = 0.161] for the cardiorespiratory fitness classification. HR was higher with caffeine than with placebo (163 ± 12 vs 157 ± 13 b**∙**min^− 1^ overall). For the neuromuscular fitness classification (Table 3), there were only significant main effects of treatment [F(1, 16) = 17.07, *p* < 0.001, ES = 0.516] and time [F(2, 34) = 14.39, *p* < 0.001, ES = 0.473]. There was no significant group effect for either classification (*p* > 0.05).

**Table 3:** Heart (HR) and rating of perceived exertion (RPE) for the cardiorespiratory and neuromuscular fitness classifications (mean ± SD)

Classification	Variable	Treatment	Group	Treadmill periods			
				1st	2nd	3rd	4th
Cardiorespiratory fitness	HR (b∙min^−1^)*†	Caffeine	HCF	152.88 ± 10.70	159.02 ± 10.62	157.49 ± 14.53	172.18 ± 6.76
			MCF	159.01 ± 11.86	163.22 ± 11.71	161.13 ± 13.33	173.30 ± 15.42
		Placebo	HCF	150.97 ± 11.99	156.84 ± 11.14	150.88 ± 12.49	160.03 ± 7.24
			MCF	151.43 ± 16.59	161.44 ± 12.28	159.50 ± 11.59	163.20 ± 20.05
	RPE*†	Caffeine	HCF	10.00 ± 1.51	11.67 ± 2.28	11.70 ± 2.29	14.67 ± 1.93
			MCF	11.23 ± 0.98	13.03 ± 1.34	13.47 ± 1.57	15.23 ± 1.66
		Placebo	HCF	10.37 ± 1.71	12.33 ± 2.15	12.97 ± 1.58	15.53 ± 1.34
			MCF	11.20 ± 1.03	13.30 ± 1.70	14.43 ± 1.49	16.60 ± 1.55
Neuromuscular fitness	HR (b∙min^−1^)*†	Caffeine	HNF	155.28 ± 13.33	163.76 ± 14.14	159.70 ± 17.36	178.54 ± 10.39
			MNF	156.61 ± 9.86	158.49 ± 6.66	158.91 ± 9.72	168.22 ± 11.73
		Placebo	HNF	155.00 ± 11.25	158.16 ± 12.42	157.70 ± 2.12	167.19 ± 10.40
			MNF	147.40 ± 16.15	160.12 ± 11.42	152.69 ± 13.07	156.04 ± 16.82
	RPE*†	Caffeine	HNF	10.53 ± 1.64	11.83 ± 1.96	11.83 ± 2.25	14.30 ± 1.16
			MNF	10.70 ± 1.17	12.87 ± 1.89	13.33 ± 1.77	15.60 ± 2.09
		Placebo	HNF	10.63 ± 1.65	12.27 ± 1.57	13.10 ± 1.56	15.47 ± 1.22
			MNF	10.93 ± 1.26	13.37 ± 2.21	14.30 ± 1.64	16.47 ± 1.60

* Significant main effect of treatment (*p* < 0.01)

† Significant main effect of time (*p* < 0.001)

### Perception of effort

There were significant main effects of treatment [F(1, 18) = 13.16, *p* = 0.002, ES = 0.422] and time [F(2, 39) = 101.83, *p* < 0.001, ES = 0.850], as well as an interaction between treatment and time [F(2, 36) = 4.58, *p* = 0.006, ES = 0.203] for the cardiorespiratory fitness classification on RPE (Table 3). RPE was lower with caffeine than with placebo (12.6 ± 1.7 vs 13.3 ± 1.6 overall), as seen in Table 3. For the neuromuscular fitness classification (Table 3), there were also significant main effects of treatment [F(1, 18) = 13.49, *p* = 0.002, ES = 0.428] and time [F(3, 54) = 107.01, *p* < 0.001, ES = 0.856], as well as an interaction between treatment and time [F(2, 34) = 3.35, *p* = 0.05, ES = 0.157] on RPE. There was no significant group effect for either classification (*p* > 0.05).

### Energy expenditure and fuel oxidation

No differences between treatments or groups were found in energy expenditure, fat oxidation, or carbohydrate oxidation (*p* > 0.05). The overall energy expenditure was 15.74 ± 1.76 kcal·min^− 1^, fat oxidation was 1.13 ± 0.19 g·min^− 1^, and carbohydrate oxidation was 1.41 ± 0.47 g·min^− 1^.

### Blood metabolites

**Fig. 3:**
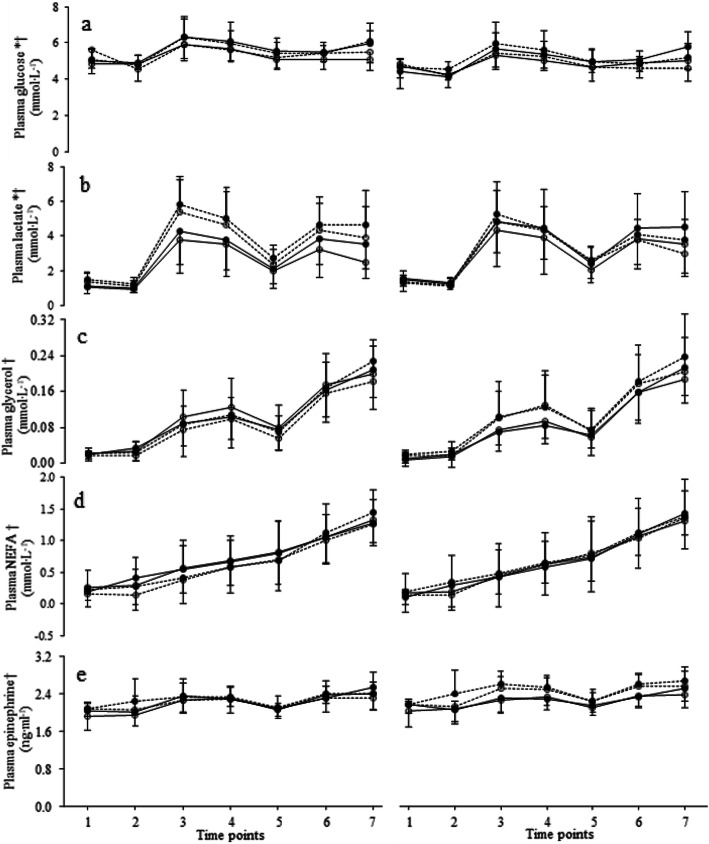
Means and SD of plasma glucose (**a**), lactate (**b**), glycerol (**c**), NEFA (**d**) and epinephrine (**e**) with caffeine (full circles) and placebo (open circles). Participants were divided according to cardiorespiratory (left) or neuromuscular fitness (right), with high groups shown with solid lines and medium groups shown with dashed lines. One to 7 correspond to time points as follows: 1, 75 min before the start of the trial; 2, 15 min before the start of the trial; 3, between the 1st and 2nd periods; 4, immediately after the end of the 2nd period; 5, immediately before the start of the 3rd period; 6: between the 3rd and 4th periods; 7, at exhaustion. *Significant treatment effect (*p* ≤ 0.01). †Significant time effect (*p* < 0.001)

When data were analyzed based on cardiorespiratory fitness level, plasma glucose (Fig. 3a) exhibited significant main effects of treatment [F(1, 18) = 14.30, *p* = 0.001, ES = 0.443] and time [F(3, 57) = 13.73, *p* < 0.001, ES = 0.433], as well as an interaction between treatment and time [F(3, 47) = 5.38, *p* < 0.001, ES = 0.230]. Plasma glucose was higher with caffeine than with placebo (overall 5.6 ± 0.7 vs 5.3 ± 0.6 mmol∙l^− 1^). Similarly, when analysis was based on neuromuscular fitness level, there were also a treatment effect [F(1, 18) = 13.15, *p* = 0.002, ES = 0.422], a time effect [F(3, 60) = 14.24, *p* < 0.001, ES = 0.442], and an interaction between treatment and time [F(3, 45) = 5.19, *p* < 0.001, ES = 0.224]. There was no significant group effect with either classification (*p* > 0.05).

Within the cardiorespiratory fitness level analyses, there were significant main effects of treatment [F(1, 18) = 8.12, *p* = 0.01, ES = 0.311] and time [F(2, 33) = 65, *p* < 0.001, ES = 0.782], as well as an interaction between treatment and time [F(3, 58) = 4.31, *p* < 0.007, ES = 0.193] in plasma lactate (Fig. 3b). As with glucose, lactate was higher with caffeine than with placebo (overall 3.3 ± 1.2 vs 2.9 ± 1.2 mmol∙l^− 1^). Within the neuromuscular fitness level analysis, there was also a treatment effect [F(1, 18) = 8.43, *p* < 0.001, ES = 0.319], a time effect [F(2, 30) = 62.12, *p* < 0.001, ES = 0.775], and an interaction between treatment and time [F(3, 61) = 4.31, *p* < 0.006, ES = 0.193].

With regard to plasma glycerol (Fig. 3c), NEFA (Fig. 3d), and epinephrine (Fig. 3e), the only statistically significant outcomes of the factorial ANOVA were a main effect of time for the cardiorespiratory fitness classification [F(2, 35) = 129.65, F(3, 45) = 116.78, and F(3, 59) = 22.54, respectively; *p* < 0.001 for all; ES = 0.878, 0.866, and 0.556, respectively] and for the neuromuscular fitness classification [F(2, 35) = 134.05, F(3, 47) = 110.06, and F(3, 51) = 20.78, respectively; *p* < 0.001 for all; ES = 0.882, 0.859, and 0.536, respectively], as well as a main effect of group on epinephrine for the neuromuscular fitness classification [F(1, 18) = 6.25, ES = 0.258, *p* = 0.022]. The time effect was due to a general increase in all three parameters from the beginning to the end of the trials, and the group effect was due to epinephrine being higher in the MNF group.

### Influence of body mass and body fat

When body mass, body fat, or both variables were added to the analysis as covariates, all statistical outcomes but one were qualitatively the same. That is, all significant outcomes of ANOVA remained significant with ANCOVA and all non-significant outcomes of ANOVA remained non-significant with ANCOVA, with the exception of NEFA, which, although not significantly different between the HCF and MCF groups according to ANOVA, became significantly different (specifically, higher in the HCF group) when body fat [F(1, 17) = 4.49, ES = 0.209, *p* = 0.049] or body mass and body fat were used as covariates [F(1, 16) = 5.22, ES = 0.246, *p* = 0.036]. These finding show that body mass and body fat had no influence on the caffeine responses.

## Discussion

The novel finding of the present study is that caffeine, at a dose of 6 mg∙kg^− 1^, was effective in improving time to exhaustion, CMJ, and RPE of male soccer players in a trial simulating the cardiovascular demands of a soccer game regardless of differences in cardiorespiratory or neuromuscular fitness. Thus, these parameters do not appear to modulate the ergogenic effects of caffeine supplementation. These findings complement those of our previous study [10], which, through a different analysis of the data from the same participants, concluded that caffeine was ergogenic in both high and low caffeine responders.

The ergogenicity of caffeine on endurance performance found in the present study cannot be attributed to metabolic effects, since substrate utilization during the final stage of the time-to-exhaustion protocol was not different between the caffeine and placebo trials. A more probable explanation is a stimulatory effect on the central and peripheral nervous systems, since caffeine crosses the blood-brain barrier and acts as an adenosine receptor antagonist, increasing central motivation to exercise, peripheral neuromuscular activation, heart rate, myocardial oxygen consumption, and blood flow [24, 25]. This hypothesis is supported by the reduction in RPE observed within all the cardiorespiratory (HCF and MCF) and neuromuscular (HNF and MNF) fitness groups of the current study following caffeine ingestion relative to placebo. It is interesting that the effect of caffeine on time to exhaustion was similar between the high and medium cardiorespiratory fitness groups. This is in agreement with the finding of similar improvement in time to fatigue between endurance trained and untrained individuals (with different VO_2_max values) following caffeine supplementation [26]. It appears that VO_2_max, which is mainly determined by cardiac output and the ability of the working muscles to take up oxygen [27], is not a determining factor for the effects of caffeine on endurance performance.

The present study revealed a CMJ improvement following caffeine ingestion relative to placebo. This effect was independent of CMJ performance. Individual differences in CMJ performance could result from interaction of multiple factors such as fiber type proportions, muscle size, speed of neural activation of the musculature, rate of force development, and maximum strength [28–30]. Based on our findings, it appears that caffeine’s effects on CMJ is independent of these factors, since both the high and medium CMJ groups showed similar improvements with caffeine ingestion. A potential explanation regarding the improvement in CMJ with caffeine is the increase in voluntary activation during isometric, concentric, and eccentric contractions, thus increasing strength and power regardless of contraction mode [31]. It has been suggested that this activation might be due to acute neural adaptations at supraspinal and cortical levels [31]. In addition, caffeine may improve excitation-contraction coupling by facilitating Ca^2+^ release from the sarcoplasmic reticulum [32] and/or improve Na^+^/K^+^ pump activity [33].

The positive effect of caffeine on plasma glucose found in the present study may indicate that caffeine stimulated liver and skeletal muscle glycogenolysis, resulting in increased release to and reduced uptake from the bloodstream during exercise [34]. Likewise, the higher plasma lactate concentration during the caffeine trials might be the result of enhanced muscle glycogenolysis, accompanied by an inability of the mitochondria to absorb the increased pyruvate production for aerobic ATP resynthesis [35]. Alternatively, the increased plasma lactate concentration with caffeine may indicate an inhibition of lactate uptake by non-exercising muscles and/or the liver [35].

This study offers novel insight into the effects of caffeine on biological responses and exercise performance relative to different fitness levels of the athletes. However, we should point out that, since our sample consisted of active soccer players, even the medium fitness groups had fairly high cardiorespiratory and neuromuscular fitness levels. Consequently, there may be a false negative for detection of differences between high and low attributes in the broader sports population. In addition, while performing a series of tests imitating a soccer game in the laboratory offers the advantage of strict control over the test variables, it should not be underestimated that results during actual game might be different due to uncontrolled variables (e.g., weather, tactics, opponents and ball skills). Another limitation is the relatively small sample size of the groups. This was imposed by the inability to find more well-trained professional soccer players and by the high cost of the analyses. Finally, although body mass and body fat did not influence the caffeine responses, we acknowledge that the stratification employed produced groups that were fairly different in body mass and significantly different in body fat. This should be controlled in the future for increased validity of the findings.

## Conclusion

The results of our study show that caffeine was effective in improving endurance and neuromuscular performance in high-intensity exercise regardless of cardiorespiratory or neuromuscular fitness level in well-trained young athletes. Hence, the ergogenicity of caffeine seems not to depend on cardiorespiratory or neuromuscular factors.

## Data Availability

All data generated or analyzed during this study are included in this published article.

## Abbreviations

CMJ: Countermovement jump
ES: Effect size
HCF: High cardiorespiratory fitness
HNF: High neuromuscular fitness
HR: Heart rate
MAP: Mean arterial blood pressure
MCF: Medium cardiorespiratory fitness
MNF: Medium neuromuscular fitness
NEFA: Non-esterified fatty acids
RER: Respiratory exchange ratio
RPE: Rating of perceived exertion
RT: Reaction time
